# Rationale and methods of a cluster-randomized controlled trial to promote active and healthy lifestyles among Brazilian students: the *“Fortaleça sua Saúde”* program

**DOI:** 10.1186/s12889-015-2543-2

**Published:** 2015-12-07

**Authors:** Valter Cordeiro Barbosa Filho, Adair da Silva Lopes, Antônio Barroso Lima, Evanice Avelino de Souza, Fabiane do Amaral Gubert, Kelly Samara Silva, Neiva Francenely Cunha Vieira, Nicolino Trompieri Filho, Thábyta Silva de Araújo, Pedro Felipe Carvalhedo de Bruin, Jorge Mota, “Fortaleça sua Saúde” Working Group

**Affiliations:** Department of Physical Education, Research Centre in Physical Activity and Health, Federal University of Santa Catarina, Florianopolis, Brazil; Research Centre in Physical Activity, Health and Leisure, Faculty of Sport, University of Porto, Porto, Portugal; Institute of Physical Education and Sports, Research Centre in Physical Activity and Health in School, Federal University of Ceara, Fortaleza, Brazil; Department of Clinical Medicine, Federal University of Ceara, Fortaleza, Brazil; Department of Nursing, Aids Project: Education and Prevention, Federal University of Ceara, Fortaleza, Brazil; Department of Education-Fundamentals, Faculty of Education, Federal University of Ceara, Fortaleza, Brazil

**Keywords:** Adolescent behavior, Intervention, Motor activity, Mediating variables, Mental health, Obesity, Adolescent health, School, Health promotion, Brazil

## Abstract

**Background:**

Interventions on adolescents’ lifestyle are important, but the main mechanisms that explain the changes (mediating variables) on lifestyle are unclear. This paper presents the rationale and methods of an intervention program focused on promoting active and healthy lifestyles (especially physical activity [PA] practice and reducing screen time) among Brazilian students-the *Fortaleça sua Saúde* program (Portuguese for “strengthen your health”).

**Methods/Design:**

This is a school-based cluster-randomized controlled trial. Three intervention and three control (no intervention) full-time public schools were randomly selected in Fortaleza, northeastern Brazil. Students (*n* = 1,272) from classes in Grades 7–9 were eligible, and 1,085 (548 in the intervention and 537 in control schools) completed the baseline and follow-up measures. The program duration was approximately four months and took place in 2014. Intervention strategies focused on teacher training, activities on health in the curriculum, active opportunities in the school environment (the availability of equipment for PA), and health education (health materials for students and parents). Data collection was undertaken before and immediately after the intervention. The primary variables included the practice of PA (weekly PA volume, PA behavior change stage and preference for PA during leisure-time) and screen time (TV and computer/video games). Potential intrapersonal, interpersonal and environmental mediators of PA and screen time were evaluated by a standardized questionnaire. Other lifestyle components (e.g., eating habits, substance use), psychological (e.g., self-rated health, body satisfaction) and biological (general and abdominal obesity) aspects, as well as academic performance were also evaluated in the total sample. Depressive symptoms, eating disorders, sleep quality, objectively-measured PA, and sedentary time were evaluated in obese students.

**Discussion:**

If effective, this program will contribute to the development of public policies for the promotion of active and healthy lifestyles in youth, especially those from low- and middle-income countries. The main intrapersonal, interpersonal and/or environmental mediators of PA and screen time may also be indicated. Finally, we anticipate that the proposed strategies may be adaptable to public schools and may even be extended to the entire school system.

**Trial registration:**

ClinicalTrials.Gov: NCT02439827. Registration date: May 3, 2015.

**Electronic supplementary material:**

The online version of this article (doi:10.1186/s12889-015-2543-2) contains supplementary material, which is available to authorized users.

## Background

An active (i.e., regular physical activity [PA] as well as low levels of sedentary time) and healthy (i.e., attitudes positively associated with health, such as healthy eating habits) lifestyle can lead to many individual and collective benefits at any age [1–4]. However, a focus on the early years has been employed due to the potential present and future health impact on the population [1–5].

Although the benefits of active and healthy lifestyles are widely reported, few young people lead an active lifestyle. International data estimates that only two out of ten adolescents meet the PA recommendations (300 min or more of moderate to vigorous PA per week) [6]. In Brazil, the proportion of students who met this recommendation fell from 43.1 % in 2009 to 30.1 % in 2012 [7]. Additionally, other unhealthy lifestyle factors are common among young people (e.g., excessive screen time and inadequate eating) [6–8]. Thus, interventions focused on promoting active and healthy lifestyles in young people are a public health priority [3, 4, 9, 10].

Systematic reviews have summarized the evidence of interventions that aimed to promote PA [10–15] and reduce sedentary behavior [5, 10, 11, 15, 16] among young people. Important implications can be identified from these reviews. First, in general, interventions had modest effect on behavior change [10, 11, 14, 16]. Second, multicomponent programs (i.e., strategies focused on different settings and individuals from the school, the family and/or the community) had more promising results on the promotion of active lifestyles [5, 10–12]. Third, the school seems to be an appropriate setting for many of the intervention strategies [10–12, 15]. Finally, the effectiveness of programs on PA and on sedentary behavior-related variables (e.g., risks and benefits, self-efficacy, social support, environmental perceptions), and how these variables (i.e., mediating variables) explain active lifestyle changes among adolescents has been little explored [13, 15].

Specifically, systematic reviews have highlighted that interventions focused on mediating variables and how they work are still scarce [5, 13, 15, 16]. For example, Van Stralen et al. [15] found 18 interventions on mediators of PA, but most of the potential intrapersonal (e.g., knowledge, self-efficacy), interpersonal (e.g., social support, peer and family model) and environmental (e.g., perception and environmental characteristics) mediators associated with PA remained largely unexplored. Evidence on the mediators of reducing sedentary behavior among young people is even more limited [5, 15, 16].

An intervention program that sought to fill the gaps was the Transform-Us! program [17]. This intervention was carried out in 2011 and 2012 for a period of 18 months and involved almost six hundred 8 to 9 year-old children from Melbourne, Australia. The intervention activities were multicomponent, including changes in physical education (PE) classes, reducing uninterrupted sedentary time and promoting health education in school. Intrapersonal, interpersonal and environmental factors related to PA and screen time were measured [17].

Results from the Transform-Us! program [18] revealed important evidence of the role played by the perceived school environment and perceived social support from teachers as mediators of PA changes among children over time. However, it is questionable whether such results would similarly occur among adolescents given the fact that they have physical, psychological and social peculiarities [19]. Additionally, the Transform-Us! program and most of the evidence from systematic reviews [5, 11, 13, 15, 16, 20] are of high-income countries. Sociocultural and structural distinctions between countries allow exploration of whether the effectiveness of interventions promoting active lifestyle and their mediators occur similarly among low-and middle-income youth populations, such as in Brazil. Considering that most of the world’s population lives in these types of countries (see the World Bank’s population estimates at www.worldbank.org/depweb), interventions in these contexts are essential in order to know what adjustments and variables are necessary for an effective promotion of active and healthy lifestyles among young people in these sociocultural situations.

Based on these assumptions, this paper presents the rationale and methods of a cluster-randomized controlled trial aiming to promote active and healthy lifestyles among Brazilian students. This program was entitled *Fortaleça sua Saúde* (Portuguese for “strengthen your health”) and included students from elementary full-time public schools (Grades 7–9) from a municipality in northeastern Brazil.

## Rationale

### Why investigate adolescents?

Adolescence — a life stage that includes those aged from 10 to 19 — makes an outstanding contribution to the individual’s current and future health [1, 4, 21]. During this period significant biological change that can affect an individual’s health status occurs [19], as well as psychological and social formation related to the adoption of risk or healthy behaviors [21].

Four main reasons explain why adolescence is a period of great interest for lifestyle studies. First, although cardiovascular disease is more common in later stages of life, the internal processes of these diseases may begin in childhood and adolescence [22]. Second, this life stage is characterized by great vulnerability to environmental factors such as the influence of the media, friends, school and the community, impacting positively or negatively on options for health behaviors [5, 23]. Third, many of the behaviors established during adolescence tend to be retained in adulthood [21, 24]. Finally, some behaviors in adolescence potentially impact on health in the early stages of life [25] and contribute to further health problems in adulthood [22].

Despite the importance of a healthy lifestyle and the dissemination of such messages in the community and wider media, data from 105 countries shows that eight out of ten 13 to 15 year-old students do not meet the PA recommendations and seven out of ten students spend two hours or more each day watching television [6]. In Brazil, the proportion of adolescents who fail to meet the PA recommendations and who watch television for two or more hours each day is about 70 % and 80 %, respectively [7]. Therefore, regional and national strategies that seek to promote an active and healthy lifestyle at this life stage are critical.

### Why investigate full-time schools in Brazil?

In general, the length of the school day in the Brazilian school model is based on classes that take place during a single period (morning, afternoon or evening separately). In terms of the curricular structure, the disciplines are based on national curriculum guidelines that include, for example, Portuguese, mathematics, science, physical education, foreign languages, and others [26]. In 2007, the Health and Education Ministries created a national program entitled *Programa Saúde na Escola (*PSE, Federal Decree No. 6286) that aims to provide comprehensive (i.e., extending the time and educational support) prevention, health promotion, and care for children and adolescents who attend schools in the public education system.

Some schools with the PSE are full-time schools. In these schools, the day begins at 7.30 am and closes at 4.00 pm, with two 20-min breaks and a lunchtime (from 11.30 am to 12.55 pm). Within the school curriculum, there is a mixture of previously mentioned disciplines from the national curricular base [26] and full-time school-specific disciplines (e.g., “Youth Protagonism” and the “Life Project”). In addition to these, “unstandardized” disciplines are offered based on the needs of the school community (i.e., teachers and students vote for disciplines that would be interesting for their school). One of the goals of the Brazilian Education Plan (Law 13,005/2014) is that a full-time education system should be offered in at least 50 % of government schools by 2024 (see www.pne.mec.gov.br/images/pdf/pne_conhecendo_20_metas.pdf).

In 2014, 165 municipal schools undertook the PSE in Fortaleza-Ceara, the fifth largest city in Brazil; 40 of these had elementary school classes (Grades 6–9). Of these 40 schools, only six were full-time. However, by 2016, the projection is that 30 of the 40 schools with PSE will be full-time (see information in www.sme.fortaleza.ce.gov.br/mapeamentoescolar).

The involvement of multicomponent and intersectoral strategies to promote healthier individuals and environments is one of the priorities of the PSE [10], as well as staff training (i.e., teachers and coordinators) to meet the health demands of Brazilian schools and communities [9, 10]. Additionally, the PSE was intended to become a comprehensive public and education policy in Brazil. Based on this, we decided to consider schools with PSE and a full-time education model as ideal environments to conduct a multicomponent program focusing on active and healthy lifestyles.

### Why is the program called Fortaleça sua Saúde?

The intervention program is called *Fortaleça sua Saúde* for the following reasons. First, due to the similarity between the city’s name (Fortaleza) and the verb *Fortaleça* (to strengthen) in its imperative form, it conveys the concept of encouragement, stimulation and excitement for students, thus corresponding with the purposes of an active lifestyle program. Second, the inclusion of the word “health” in the program’s name allows the students to identify the general purpose of the intervention program on the different aspects of a healthier lifestyle.

**Fig. 1:**
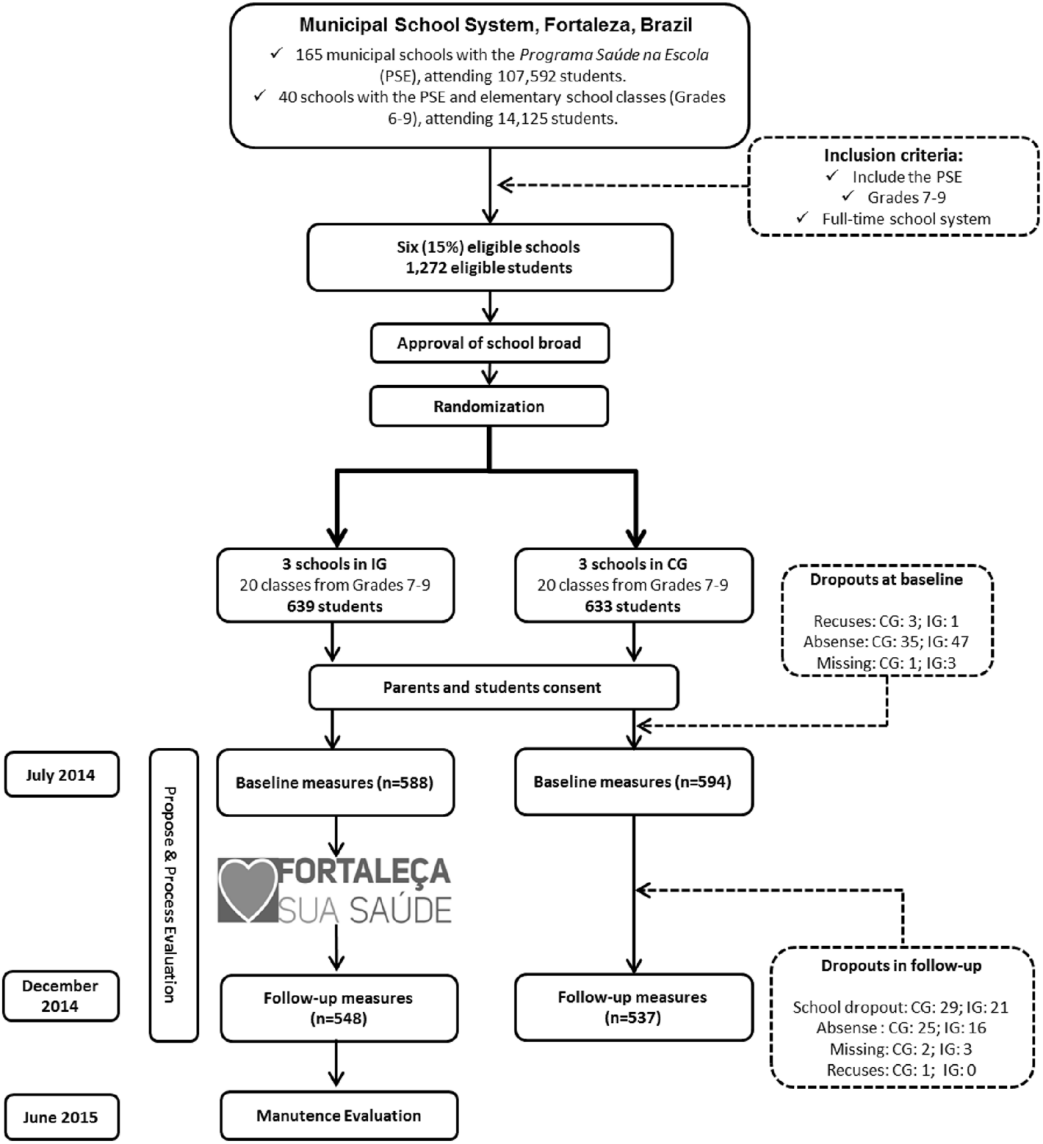
Flowchart of the *Fortaleça sua Saúde* program study. PSE: Programa Saúde na Escola; IG: intervention group; CG: control group

With regard to the program’s logo, the symbol for *Fortaleça sua Saúde* was represented by a square, symbolizing something that transmits strength as a synonym for force. As students are the population of interest, a heart was used as a widely recognized symbol of health. The program’s logo is presented in Fig. 1.

## Methods

### Aim and study design

This study was descripted following the Consolidated Standards of Reporting Trials (CONSORT) recommendations (Additional file 1). This is a cluster-randomized controlled trial and the school was the sample selection unit [27]. The primary objective of the *Fortaleça sua Saúde* program was to determine whether a multicomponent program (teacher training and activities on how to discuss health topics in the general curriculum and PE classes, active opportunities in the school environment and health education in the school community) would increase PA practice and reduce screen time among full-time elementary students (Grades 7–9) in Fortaleza, northeastern Brazil. A further primary objective was to identify the effectiveness of the program on intrapersonal, interpersonal and environmental variables related to PA and screen time, and to determine which of these variables mediate active lifestyle changes. The program’s secondary objectives included: (i) to determine whether the program is also effective for different health factors (e.g., nutritional status, health behavior, quality of life, and others); (ii) to identify its effectiveness for addressing eating disorders, sleep quality and objectively-measured PA and sedentary time among obese students; and (iii) to evaluate the program execution, including indicators of the implementation, maintenance, and costs of this program.

The participation of the students involved in this study was authorized by the parent/guardian, by signing the informed consent form. The National Research Ethics System (protocol No. 17366313.9.0000.0121) approved this research project and it follows resolutions 196 and 251 of the National Health Council. Each school will receive a final report containing the program results and practical recommendations for application in schools.

### Setting and population

Fortaleza is the capital of the state of Ceara, northeastern Brazil, and has a population of 2,452,185 inhabitants (the fifth largest city in Brazil in terms of population) [28]. This municipality has an area of 314 km^2^ and a Human Development Index (HDI) of 0.754 — it is the 19^th^ HDI among the 27 Brazilian state capitals [28]. The city of Fortaleza is geographically divided into six administrative regions [28].

According to the Municipal Education Department, Fortaleza had 464 municipal schools in 2014 (www.sme.fortaleza.ce.gov.br/mapeamentoescolar). Of these, 161 schools included classes from Grades 6–9 (34.7 %) and 165 schools included the PSE (35.5 %). Forty schools included Grades 6–9 and PSE, and six out of these schools had a full-time education model. Approximately 2,500 students were enrolled in these six full-time schools, of which 1,272 were enrolled in classes from Grades 7 to 9 (Fig. 1).

Grade six classes were substantially composed of students aged 10 and 11 years old. Based on a validation of the study’s instruments, students from the 6^th^ Grade would have difficulty reporting reliable information. Additionally, age groups considered appropriate by the World Health Organization (WHO) for studies on health behaviors are primarily 13–15 year olds [6–8] and these would be included in successive series, therefore, 6^th^ Grade students were not included.

Thus, all six full-time schools with PSE were eligible. Three schools were in the intervention condition and three additional schools received no intervention (control). The inclusion criteria were students of both genders, aged 12–15 years who were enrolled in Grades 7–9, attending full-time public schools in Fortaleza, northeastern Brazil (Fig. 1).

### Recruitment of schools and students

The six schools had similar characteristics (e.g., size, target audience, curriculum, etc.) and were located in different administrative regions (geographically dispersed). Therefore, we did not perform peers of schools during random selection.

After authorization of the study by the Municipal Education Department, all directors of eligible schools were informed about the study and the participation criteria. All directors agreed to participate without being informed which treatment group the schools would be assigned to in the study. Thus, we performed the random selection of three schools to each condition (intervention or control). Schools were visited to explain the objectives of the study and the program logistics during the semester. The neighborhood-HDI [28] where the six schools were located was similar between groups (neighborhood-HDIs of 0.215, 0.341 and 0.443 for intervention schools, and 0.170, 0.377 and 0.491 for control schools).

Of the 1,272 eligible students (639 in intervention and 633 in control schools), 1,182 students filled out the baseline measures (92.0 % and 93.8 % of eligible students from intervention and control schools, respectively). The main reason for non-participation at baseline was being absent from school on data collection days. Post-intervention data collection included 1,085 students (response rate of 93.2 % and 90.4 % in intervention and control schools, respectively). Dropping out of school was the main reason for non-participation in post-intervention data collection (Fig. 1).

### Intervention

#### Pilot study

The *Fortaleça sua Saúde* working group met during 2013 and 2014 to select intervention strategies and plan how to adapt them to the structural, material and human reality of Fortaleza’s schools. College teachers and students of different courses were involved in working subgroups for each intervention component. Following this step, the development of program material (e.g., flyers, games, banners) was undertaken.

In 2014, a pilot study was conducted in a public school. In general, we noted that many of the actions were suitable for the school environment. However, some strategies, such as the distribution of school equipment to students, needed adjustment. For this strategy, the use of a control chart was needed to prevent loss or damage to equipment. Furthermore, the rules of the games needed to be simplified and to be feasible. Finally, we noted that the activities proposed in the manuals needed tailoring to the reality and characteristics of each class and school. These aspects were discussed in the *Fortaleça sua Saúde* working group and were considered and set in the implementation of the main study program.

### Theoretical basis of the *Fortaleça sua Saúde* program

The *Fortaleça sua Saúde* program was structured to consider different theoretical aspects. Socioecological theory [29] emphasizes that intrapersonal (e.g., knowledge, self-efficacy), interpersonal (social support, peer and family models), and environmental aspects (perception and environmental characteristics) are independently and interactively influential on an individual’s behavior. The actions of the program *Fortaleça sua Saúde* program were directed to address these different levels (see Table 1). The concept of the Health Promoting in Schools framework [9, 10] was also used. Three characteristics of this framework were used in order to choose the program components: health education topics in the formal school curriculum, health values, attitudes and opportunities promoted within the school, and schools seeking to engage with families, outside agencies and the wider community [10] (see Table 1).

**Table 1:** Description of the components, characteristics, focus on potential mediators and executive agent of *Fortaleça sua Saúde* program strategies

Component descriptions and strategies	Specific focus on PA or screen time use mediators^a^	Executor agent
Training and activities in general curriculum (*Aim*: to train and encourage teachers to discuss health topics in the classroom)		
➢Training with certification focused on health topics and dynamics in the curriculum	√ *Primary*: EP (teacher support and modeling) and ENV (school)	√ Program members
➢Supplemental manual with proposed activities on health topics to be applied in the classroom	√ *Primary*: IP (knowledge, risks/benefits, types) and ENV (school)	√ Teachers
	√ *Secondary*: EP (teacher support and modeling)	
➢Interactive media for teachers to disseminate ideas and implementation of activities in classroom	√ *Primary*: EP (teacher support and modeling) and ENV (school)	√ Teachers/Program members
➢Exposition of materials (posters, murals) to disseminate health messages in school (integrated with health education)	√ Primary: ENV (school)	√ Teachers
	√ *Secondary*: IP (knowledge, risks/benefits, types, self-efficacy) and EP (peer and teacher support and modeling)	
Training and activities in PE classes (*Aim*: to train and encourage teachers to discuss topics on PA and health in PE classes)		
➢Training with certification focused on health and active dynamics in PE classes	√ *Primary*: EP (teachers support and modeling) and ENV (school)	√ Program members
➢Supplemental manual with proposed activities on active and health topics to be applied in the classroom	√ *Primary*: IP (knowledge, risks/benefits, types)	√ Teachers/Program members
	√ *Secondary:* EP (peer and teacher support, modeling) and ENV (school opportunities, school and neighborhood perceptions)	
➢Production of material by students (e.g., posters, photos) to be exhibited at school, and health events (integrated with Health Education)	√ *Primary*: IP (attitude, risks/benefits, types, self-efficacy)	√ Teachers/Program members
	√ *Secondary*: EP (peer and teacher support) and ENV (school environment opportunities, school and neighborhood perceptions)	
➢Staff support during PE classes	√ *Primary*: EP (teacher support and norms) and ENV (school)	√ Program members
Active opportunities in the school environment (*Aim*: to promote structural spaces and materials in school for PA practice, reducing sedentary time and health information).		
➢Two 10–15 min supervised sessions per week of dynamic activities during free-time in school	√ *Primary*: IP (types), EP (peers support, norms and modeling) and ENV (school opportunities, school perception)	√ Program members
	√ *Secondary*: IP (knowledge, risk/benefices, self-efficacy)	
➢Equipment for games (e.g., mini-courts, “Squash in Health”) with active opportunities and health messages during free-time	√ *Primary*: ENV (school opportunities, school perception) and EP (peers support and modeling)	√ School manager/students
	√ *Secondary*: IP (risk/benefices, types, self-efficacy)	
➢School equipment (balls, rackets, etc.) available to students during free-time in school	√ *Primary*: ENV (school opportunities, school perceptions) and EP (peer and teacher support and modeling)	√ School manager/students
➢Banners with games rules, material use guidelines and motivational and health messages (integrated with Health Education)	√ *Primary*: ENV (school opportunities, school perceptions)	√ School manager/Program members
	√ *Secondary*: IP (knowledge, risks/benefits) and EP (social support and norms)	
Health education in school community (*Aim*: to promote health knowledge to the school community, especially students and their families)		
➢Pamphlets to students in the classroom or schoolyard: 1) PA and health; 2) screen time use and health; 3) eating behaviors	√ *Primary*: IP (knowledge, attitude, types and health recommendations)	√ School manager/Teachers
	√ *Secondary*: EP (social and family support) and ENV (opportunities in school and home)	
➢Pamphlets to parents in meetings or visits to schools: 1) PA and family; 2) screen time use and family	√ *Primary*: EP (social and family support, norms and modeling) and ENV (opportunities in school and home)	√ School manager/Teachers
	√ *Secondary*: IP (risks/benefits, types and health recommendations)	

*PA* Physical activity, *PE* Physical education

^a^Potential PA and reducing screen time mediators that are included in the Socioecological and Health Promotion School frameworks and were focused during these strategies: IP: intrapersonal mediators (e.g., knowledge, types of PA or screen time, risks and benefits, self-efficacy, perceived barriers); EP: interpersonal mediators (e.g., peers, teachers and parents modeling, support and norms); ENV: environmental mediators (e.g., family environment, school environment and environmental)

Other theoretical aspects were considered for measurement of the study variables. Socio-cognitive theory indicates that there are both intrinsic and extrinsic factors associated with individual behavior [30]. Transtheoretical theory emphasizes behavior during the stages of change [31], and socioecological theory [29], highlights the role of the environment on an individual’s behavior. Thus, these aspects were also considered in the planning of intervention strategies and definitions of the response-variables and mediating variables (see Tables 1 and 2).

**Table 2:** Measured variables in the *Fortaleça sua Saúde* program

Dimension	Variables (reliability)^b^	
Primary Variables^a^		
PA	√ Weekly volume in moderate to vigorous PA and PA levels (ICC = 0.71)	
	√ PA-related behavior change (ICC = 0.76)	
	√ Preference for PA in leisure-time (ICC = 0.75)	
	√ Active commuting (walking/cycling) to school (ICC = 0.89)	
Potential PA mediators	√ *Intrapersonal:* Attitudes, self-efficacy and expectations (risks/benefits) (α range = 0.77–0.81)	
	√ *Interpersonal:* family, friend and teacher support (α range = 0.84–0.90)	
	√ *Environmental:* Neighborhood and school perception (α range = 0.61–0.78)	
Screen time	√ Daily time watching TV on weekdays and weekends days (ICC = 0.72 and 0.56)	
	√ Daily time using computer/video games on weekdays and weekends days (ICC = 0.80 and 0.75)	
	√ Reducing TV watching-related behavior change stage (ICC = 0.80)	
	√ Reducing computer/videogames using-related behavior change stage (ICC = 0.78)	
Potential reducing screen time mediators	√ *Intrapersonal:* attitude, self-efficacy and expectations (risks/benefits) (α range = 0.64–0.85)	
	√ *Interpersonal:* family modelling, support and norms (α range = 0.56–0.78)	
	√ *Environmental:* family and school environment, house and bedroom characteristics (α range = 0.73–0.85)	
Secondary variables^a^	√ Body mass index	√ Self-rated health (ICC = 0.90)
	√ Waist circumference	√ Stress perception (ICC = 0.79)
	√ Eating habits (ICC range = 0.71-0.89)	√ Body image (ICC = 0.85)
	√ Alcohol use (ICC = 0.71)	√ Sleep quality and duration (ICC range = 0.59-0.75)
	√ Tobacco use (ICC = 0.99)	√ Sleepiness (α = 0.62)
	√ Condom use (ICC = 0.98)	√ Academic performance
	√ Quality of life (ICC = 0.78)	√ School attendance
Descriptive variables^a^	√ Age (ICC = 0.99)	√ Mother’s schooling (ICC = 0.92)
	√ Gender (Kappa = 1.00)	√ Student’s occupational status (Kappa = 0.90)
	√ Father’s schooling (ICC = 0.86)	√ Family’s economy class (ICC = 0.93)
Subsample (obese) Variables^a^	√ Depressive symptoms (α = 0.90)	√ Objectively-measured PA
	√ Eating disorders (α = 0.80)	√ Objectively-measured sedentary time
	√ Sleep quality (α = 0.83)	
Evaluation variables	√ Interest of the school community for the program proposal (before baseline)	
	√ Visibility of the program during implementation (during the program)	
	√ Execution process of the strategies (during the program)	
	√ Interest in keeping the strategies in the future (immediately after the end of the program)	
	√ Maintenance of the program strategies (six months after the end of the program)	
	√ Start-up and operational costs (economic evaluation)	

*PA* Physical activity

^a^All these variables will be measured at baseline and immediately after the intervention

^b^Reliability of the self-reported measures were evaluated using Kappa’s index for dichotomous variables (e.g., gender), intra-class correlation coefficient for ordinal variables (e.g., economic class) and Cronbach’s alpha for scales (e.g., attitude scales). This measures were obtained using a sample (*n* = 194) of students who were not enrolled in the *Fortaleça sua Saúde* program

**Fig. 2:**
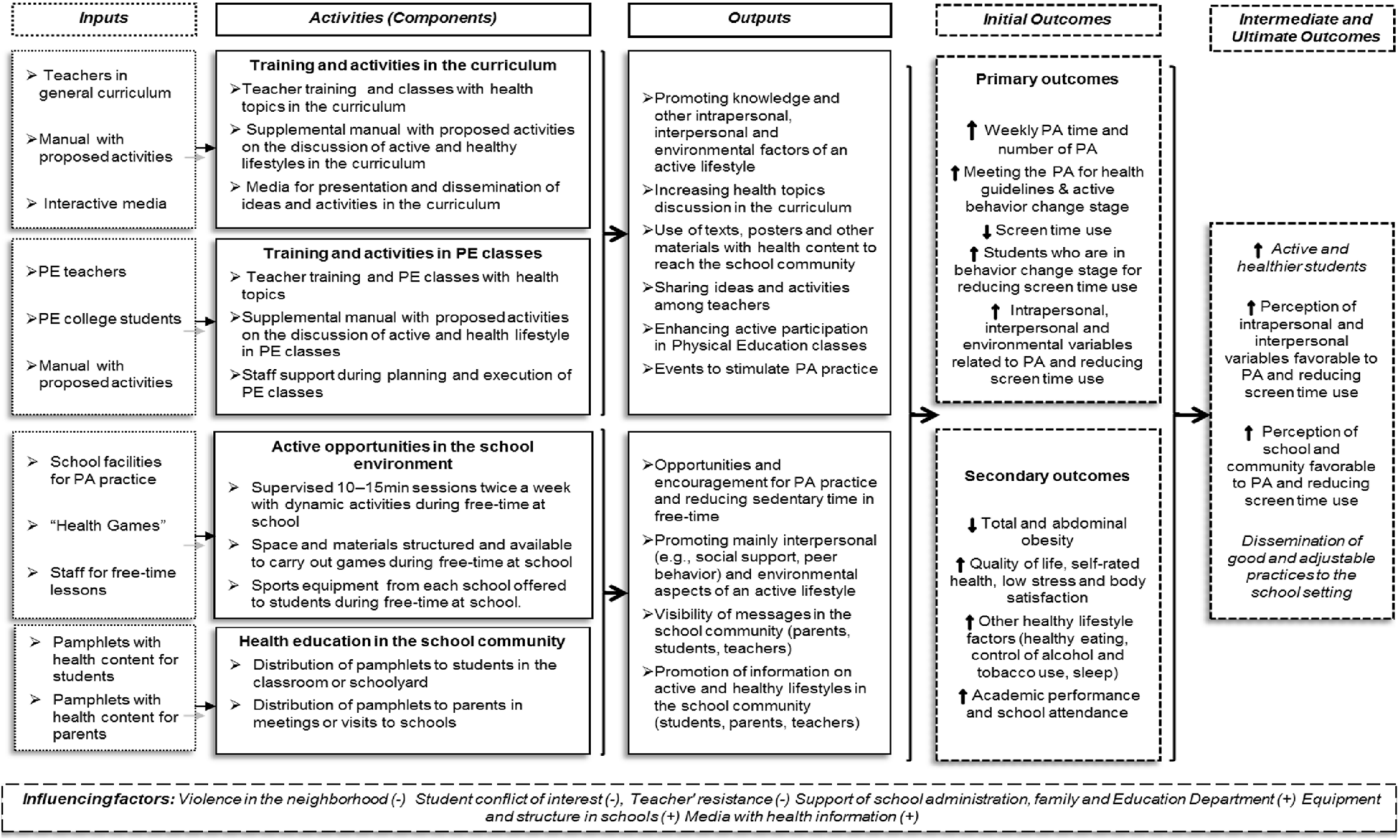
Logic model of the *Fortaleça sua Saúde* program study. PA: physical activity; PE: Physical Education

The logic model of the *Fortaleça sua Saúde* program is shown in Fig. 2 and was based on international guidelines [32]. The intervention program was structured into four main components: (i) training and activities in the general curriculum; (ii) training and activities in PE classes; (iii) active opportunities in the school environment; (iv) health education in the school community. The logic model presents inputs (resources) and the activities related to each component. Some outputs and expected results, which describe the interaction between inputs and outputs, were highlighted in interrelated boxes between the components. All resources produced in this program can be accessed by contacting the author of this article.

### Description of the intervention

The *Fortaleça sua Saúde* intervention took place during the second semester (over approximately four months) in 2014. Table 1 presents the components, activities, the focus of action in PA and screen time mediators, and application agents of the strategies.

### First component: training and activities in the general curriculum

All teachers from the three intervention schools were invited to participate in training and to execute class activities focused on the discussion of active and healthy lifestyles. Full training was structured in three stages (two direct-and one distance-learning stage) with certification recognized by the University. In order to increase teachers’ attendance, the direct-learning stages were structured to occur within the teachers’ schedule.

The first stage was a four-hour training input that took place at the beginning of the school semester. There were discussions of primary health concepts and the importance of these issues including the relationship between health, school and academic performance. Proposals for cross-cutting issues including the National Curriculum Standards [26] were also discussed. Finally, strategies directed at combining teaching tools (e.g., homework, test presentations) and health issues (e.g., PA, quality of life, healthy eating) were discussed. This training session was conducted by program members in a participatory manner and included active, dynamic, small group discussions and presentations in order to enhance content absorption and to help create new classroom strategies focused on health issues [9].

Teachers received a supplemental manual in order to help with classroom activities. This manual was prepared by program staff based on other productions on health in schools (e.g., www.movebrasil.org.br; www.nchealthyschools.org; www.take10.net; www.letsmoveschools.org), and adapted specifically for Fortaleza’s schools and students. Brazilian documents on education were consulted including the Learning Expectations Municipal Education Department (www.sme.fortaleza.ce.gov.br/educacao) and the National Curriculum Standards on health topics [26]. Ministerial reports on health and education were also consulted (obtained from www.bvsms.saude.gov.br). The manual included proposals for activities according to knowledge areas (i.e., languages, social sciences, natural sciences and mathematics). For example, in mathematics, there was a proposal about teaching quantities and measures using body measurements, energy expenditure in PA or energy consumption in meals. Each proposal included a description, required materials, alternative observations, and supporting texts. Teachers who were unable to attend the training also had access to the manual and could participate in other training stages.

Teachers were encouraged to undertake the activities or to create and implement similar strategies in the classroom during the semester. In general, the activities performed in the classroom included text production, production and exposition of videos, posters and/or booklets (newsletters or flyers) on different health issues. The themes in the manual were suitable for the interests and expertise of teachers and students as well as for the structural and material conditions of each school. A social page was created specifically to monitor, assist and promote activities among teachers from the intervention schools. Finally, teachers from each school met at the end of the semester to discuss implementation of these activities.

### Second component: training and activities in PE classes

We conducted a four-hour training tailored to PE teachers at the beginning of the school semester. They were instructed to structure predominantly active PE classes, even in classes with a theoretical content. For example, a class focused on PA types and the relation between PA and a healthy diet would be structured in active lessons. We chose this because the active time in PE classes can be a beneficial factor to total PA and adolescent health [12, 20].

A supplemental manual with lesson plans and handouts was also developed and distributed to teachers. This manual was built for the program staff and was organized by grades (Grades 7–9). Materials from the *Educação Física* + Project [33], the PE-specific National Curriculum Standards [26], and health issues (www.bvsms.saude.gov.br) underpinned the manual’s content. The manual included four units: (i) PA and health (e.g., PA and leisure, cooperative games, PA with parents); (ii) health factors (e.g., sedentary time, diabetes and hypertension, quality of life); (iii) sports (e.g., athletics, volleyball, functional training, combat sports); and (iv) popular games (e.g., games, dancing and adventure sports). The adolescents’ production of posters and text with health issues content was completed either as classwork or as homework.

An undergraduate PE student supported all PE classes (20 classes with two PE lessons each week) during the semester. These staff collaborated in the planning of activities, material procurement and implementation of lessons. Additionally, one PE-related event (e.g., dance festival or games competition) was organized in order to combine PE classes and school cultural events (e.g., Student’s Week) and to encourage the active participation of students during school events.

### Third component: active opportunities in the school environment

These intervention strategies aimed to promote opportunities for PA practice within the school and to encourage it during out-of-school time, as well as to disseminate information on the importance of an active and healthy lifestyle [10, 11, 20]. Supervised and unsupervised activities were considered [11, 14]. Some of these strategies were focused on the engagement of girls — a high-risk subgroup for inactive lifestyles [6, 8].

Supervised 10–15 min sessions, called “Gym in School”, were performed twice a week. These sessions were composed of physical (e.g., stretching, located exercises) or dynamic (e.g., games and rhythmic activities) activities in small and large groups in order to involve young people in PA during free-time at school. A member of staff conducted these sessions in a variety of open spaces in the school (e.g., school entrance, courtyard, court).

Space and equipment were structured and made available to play games in free-time during the school day. Games were created by the program team based on the purpose of the intervention [10, 14, 20], and were tailored to the school environment and the PA interests of the young people (as identified in the pilot study). In each school, two small courts were painted on the school courtyard to be used for different games (e.g., foot- /volleyball and other popular games). Additionally, markings were made in the school gymnasium for use in games with several groups of students at the same time. A game called “Squash for Health” was created with positive and negative pictures of PA and screen time (e.g., cycling or watching television). During this game, points were gained on positive images and lost on negative images. A game called “Active Board” was set on a school wall because this game needs to be played on foot (i.e., to reduce the sitting time) during breaks. In groups of 4 to 6 participants, students threw a large die and would have to perform the corresponding activities indicated on the board (e.g., spend 20 s jumping on one leg) to complete the game.

An inventory of sports equipment (balls, rackets, nets, etc.) at each school was undertaken and the school coordinator was consulted about enabling students to have access to the equipment during their free-time at school. The school coordinators also released some PA equipment for the students’ use during school breaks. A control sheet was used to record the equipment used by the students.

Staff implemented these strategies in the first two weeks of the semester in order to guide the execution, access to facilities, and understanding of the rules of the games. During the semester, some PE classes and Gym in School sessions incorporated these games in order to encourage students to play them during free-time at school. Explanatory banners were displayed in schools explaining game rules and access to equipment. Motivational messages, for example, “Let’s play with friends!” and health messages, for example, “Practicing PA with friends is very good for the health!”, were also included on the banners.

### Fourth component: health education in the school community

Some of the previous strategies had an additional focus on promoting awareness about the benefits of an active and healthy lifestyle; principally, the materials produced in the classroom in general and PE classes and the banners exposed in schools (see Table 1).

Additionally, pamphlets with messages about active and healthy lifestyle were distributed in intervention schools. The program team designed these pamphlets based on other program materials (e.g., www.movebrasil.org.br, www.nchealthyschools.org; www.take10.net; www.letsmoveschools.org). The content was adapted to the program objectives and local circumstances. Teachers and professionals specializing in health education for young people selected the content and format of the health messages. Following this, marketing experts from the Municipal Education Department designed and produced the pamphlets.

Three pamphlets were directed at students: (i) PA and health; (ii) screen time and health; and (iii) healthy eating and healthy behaviors. Two pamphlets were directed at parents: (i) PA and parents/the family; and (ii) screen time and parents/the family. In each pamphlet, messages were also directed at intrapersonal (e.g., knowledge, self-assessment), interpersonal (e.g., social and family support), and environmental (e.g., supportive environments for healthy habits) factors (see Table 1).

The pamphlets were delivered during the first month of the program to a member of the school administration (coordinator or director). Guidelines were provided on the objectives of the pamphlets and their distribution. In general, the pamphlets focused on students were delivered early in the school day or during classes. The pamphlets focused on parents were delivered during parents’/teachers’ meetings or during parental visits to school. Teachers were encouraged to use the pamphlets during lessons that involved health messages.

### Control schools

Schools from the control group underwent one semester with the regular and conventional activities of a full-time school. In general, the control schools had two weekly PE classes that included content and activities according to the perspective of their teachers. Conventional PSE also took place in these schools.

### Outcome measures and evaluations

#### Data collection

The initial data collection was carried out before the start of the intervention (during two weeks in July 2014). A further data collection that incorporated the intervention phase took place immediately after the semester (two weeks in November and December 2014). Data collection occurred over two or three days in each school in order to include eligible students. Evaluators who had previously been trained in theoretical explanations and practical simulations administered the questionnaires. Some evaluators knew which schools were in the intervention and control treatments because they were enrolled in the intervention implementation; training was focused on the standardization of data collection independent of the condition to which the schools had been assigned in the trial. The questionnaire was administered to students in the classroom, without the presence of teachers. During administration the evaluators provided instructions and read each question aloud. The students then answered the questions. Other evaluators helped the students to answer the questions. Health professionals (PE and nursing) took anthropometric measures (weight, height and waist circumference).

Instruments validated for, or adapted to Brazilian young people were used to measure the study variables. Reliability of the self-reported measures was evaluated using Kappa’s index for dichotomous variables (e.g., gender), intra-class correlation coefficient for ordinal variables (e.g., economic class), and Cronbach’s alpha for scales (e.g., attitude scales). These reliability measures were obtained using a sample (*n* = 194) of students who were not enrolled in the *Fortaleça sua Saúde* program (see measures in the Table 2).

### Primary variables

#### PA

A list of 24 types of PA validated for Brazilian adolescents [34] was used to measure PA practice. This instrument allows students to report the weekly frequency and the daily duration of each PA they perform in a typical week. Thus, the weekly PA volume and PA levels can be estimated [3]. The PA-related behavior change stage was also evaluated using a question proposed by Marcus et al. [31]. The preference for PA in leisure-time (e.g., sports, games, dance, etc.) and the mode of traveling to school (car, bus, bicycle, on foot) was evaluated [35, 36].

### Screen time

Screen time during leisure-time was assessed using four items. These questions were related to habitual use of each screen time behavior (watching television, using the computer/video games) on weekdays and weekend days separately. These items have been used to measure screen time among adolescents from several countries [6, 8] including Brazil [7, 36]. The behavior change stage to reduce screen time was also evaluated using the criterion of a behavior change stage of two hours daily [37]. Every student answered two questions on the behavior change stages for reducing television watching or computer/video game use [37].

### Intrapersonal, interpersonal and environmental variables associated with PA

A validated instrument for Brazilian adolescents [38] was used to measure intrapersonal, interpersonal and environmental variables related to PA practice. The validation process included item selection based on international instruments, translated and content validated by experts, as well as a study of test-retest application (factorial validity and reliability) [38].

The questionnaire consisted of five scales and responses were recorded on a four-point Likert scale for PA-related variables: attitude (5 items), expectations (10 items), self-efficacy (12 items), perceived neighborhood environment (16 items), parental (6 items), and friends’ support (6 items). The program was focused on the school environment and, therefore, we decided to include two other scales. A teacher’s support scale (6 items) was built considering the structure of the items on the parental and friends’ support scales [38]. A perceived school environment scale (6 items) was built on PA-related school aspects that were identified in a systematic review [39].

### Intrapersonal, interpersonal and environmental variables associated with screen time

A questionnaire was developed and validated in order to measure intrapersonal, interpersonal and environmental variables associated with screen time among adolescents. This instrument was based on the socioecological theory [5] and considered similar questionnaires [37, 40–43] and a systematic review [5] of the item selection. The selected items underwent a process of translation and back translation and adjustments for cross-cultural adaptation (local culture and terms). Subsequently, five researchers experienced in validation tools and screen time evaluated the questionnaire and indicated adaptations and other potentially relevant items. Finally, a test-retest study with an independent sample was performed.

The questionnaire included nine four-point Likert scales. Three intrapersonal (attitude [3 items], expectations [12 items] and self-efficacy [11 items]), three interpersonal (family and friends modeling [4 items], social support [4 items] and family norms [6 items]) and three environmental (family environment [8 items], school environment [4 items] and environmental characteristics [8 items]) variables were considered from this instrument.

### Secondary variables

Secondary variables were evaluated in order to identify the impact of the *Fortaleça sua Saúde* program on other health-related factors (e.g., nutritional status, health behaviors) and academic performance (Table 2).

Anthropometric assessments included the measurement of body weight (kg), height (cm), and waist circumference (cm), and the calculation of body mass index (weight [kg]/height^2^ [m^2^]). International standardization was considered [44].

Health perception and quality of life were measured using two items from the World Health Organization Quality of Life [45] instrument. The question, “How would you describe the level of stress in your life?” [36], was also applied to measure stress levels.

Body image was obtained by self-assessment on the nine-silhouettes scale proposed by Stunkard et al. [46]. Considering these silhouettes, three questions were asked about the perception of current physical appearance, healthy body image, and ideal body [46].

Eating habits were evaluated using three items related to healthy foods (fruit juice, fruit and vegetables) and three unhealthy foods (soft drinks, savory foods and sweets) in a typical week [7, 35, 36]. Two items on the frequency of tobacco and alcohol use in the month preceding the survey, and a question on condom use in the year preceding the survey were included [7].

Two items on habitual sleep duration and sleep quality were used [36]. Additionally, the evaluation of sleepiness (i.e., chances of dozing or sleeping) in eight students’ living situations (e.g., studying, talking, inactive commuting to school) was considered [47].

Academic performance (standardized tests and academic achievement) and school attendance were evaluated [48]. The notes and standardized scores of the students were obtained from the schools and organized by semester to indicate the pre-intervention period (the first semester of 2014) and during/post-intervention (the second semester of 2014).

### Subsample of obese adolescents

In order to meet one of the secondary objectives of the program, students who were obese [49] were included in a specific assessment for the subsample. The presence of depressive symptoms was evaluated using the scale proposed by Silveira and George [50]. Eating disorders were assessed using the Eating Attitudes Test (EAT-26), validated for Brazilian adolescents [51]. Finally, sleep quality was measured using the Pittsburgh Sleep Quality Index (PSQI) [52].

Obese adolescents wore ActiGraph GT1M accelerometers on the left wrist for seven consecutive days. Epochs of five-seconds were programmed. Procedures for data validation and cut-off points (in counts/min) to determine the time spent in sedentary, light, moderate, and vigorous activities were based on a previous publication [53].

### Control and descriptive variables

Potential confounders were measured and included in the data analyses (Table 2). Students’ occupational status (working; not working) was measured [36]. Economic class and parents’ schooling were evaluated with the questionnaire of the Brazilian Association of Research Companies [54]. This instrument groups subjects into economic classes (A1 [richest], A2, B1, B2, C1, C2, D, and E [poorest]) based on a score combining ownership of assets, parents’ schooling and number of employees in the household.

### Evaluation plan

Following recommendations for evaluation in PA interventions [32], the *Fortaleça sua Saúde* program evaluation was performed by members who were not enrolled in the program design and implementation. The evaluation team was followed by specialists with experience in health education and evaluation. The implementation process, program visibility, and interests of the school community (students, teachers and parents) before and after the program implementation were considered [32]. Initially, the opinions of teachers, students and coordinators on the importance, ideas and acceptance of the implementation of a health program at their school were collated. During the program, members of the evaluation team visited the schools in order to identify the implementation process of the strategies as well as to assess the program visibility in the school environment. Immediately after the program, teachers, students and coordinators reported satisfaction with the strategies and their continuity prospects for the following semesters. Six months after the program end (June 2015), the evaluation team will observe the continuity of intervention strategies. This data collection will involve independent samples and instruments in order to answer each evaluation aim.

An economic evaluation of the intervention was included in the evaluation plan. This incorporated the start-up and operational costs of the intervention delivery, both monetary and nonmonetary inputs. The intervention costs were assessed on the basis of the attendance lists, registration forms, and project logbooks of the researchers and school professionals.

### Statistical considerations

All sample size power calculations considered a statistical power of 80 % and a 5 % significance level for two-tailed tests. An odds ratio of two [35] was considered, that is, an odds ratio of two students from the intervention group becoming active after the program compared to their peers from the control group (www.openepi.com). Therefore, we estimated a sample of 480 subjects (1:1 between intervention and control groups). A cluster sample selection procedure can involve biases [35] and we, therefore, decided to duplicate the study sample for 960 schoolchildren. Additionally, considering a margin of 20 % for possible losses and refusals, ~1,200 students would be a sufficient sample size to detect a moderate-sized effect with 0.80 of power.

Moderate mediated effect sizes (structural equation models) of the intervention on the primary outcomes were expected (effect size of 0.30). We considered approximately 70 observed variables and the development of up to ten latent variables, considering the number of items included in the PA and screen time questionnaires. Thus, the recommended minimum sample size was 400 adolescents (www.danielsoper.com/statcal); a doubled sample due to the cluster sample selection procedure [35] and an anticipated 20 % dropout rate during the intervention estimating a sample of 960 students. Hence, ~1,200 students would be a sufficient sample size to detect a moderate-sized effect with 0.80 of power for the mediating variables.

We will test whether randomization resulted in a balanced distribution of variables between control vs. intervention students, as well as participating vs. dropout students. The variables that have differing distributions between the two groups will be entered as confounders in all models that test the effectiveness of the *Fortaleça sua Saúde* program.

The effect of the *Fortaleça sua Saúde* program on the primary variables will be tested in accordance with the intention-to-treat principle and in a completers-only framework using Mplus [55]. Intention-to-treat and completers-only analyses will be performed in order to identify the effect of the intervention condition in comparison to the control condition.

Because the data have a multilevel structure (i.e., individuals are clustered within a school), the individual respondents may not necessarily be independent within each school. To correct this, the procedure for complex samples in Mplus will be used in relation to the school. Finally, the results will be reported in accordance with the Consolidated Standards of Reporting Trials (CONSORT) [56].

Statistical procedures proposed by MacKinnon and Dwyer [57] will be considered to identify the effectiveness of the program according to the primary variables and mediating variables, as a common method of intervention study on PA and screen time [13, 15]. Initially, changes in the primary variable (e.g., changes in weekly PA time) will be considered in a regression under the influence of the independent variable (intervention group). In the second model, the mediating variables will also be included in the regression model. Thus, the value of the mediated effect will be calculated by the difference coefficient of the independent variable between the two models [57]. The measure of effect will be expressed in standardized scores (beta) or as an odds ratio, depending on the nature and manner of treatment of the primary variables.

Regression models will also be built in order to identify the effectiveness of the program on secondary variables (e.g., body image, eating habits). The effectiveness of the program on variables among obese students will be analyzed separately using the same statistical procedures. The implementation and economic evaluation will be qualitatively and quantitatively analyzed, considering the different indicators of the evaluation of PA interventions [32]. All analyses will be built using the Mplus program and will be adjusted for possible confounding variables (behaviors at baseline and control variables). The level of significance for the study will be 5 % for two-tailed tests.

## Discussion

The *Fortaleça sua Saúde* program aimed to identifying whether the combination of discussions on health topics in the curriculum, structural changes in the school environment, and health education in the school community can increase PA practice, reduce screen time and change potential mediators of these variables among students. One of the strengths of this program was considering strategies specific to PA and screen time. Sedentary behavior and physical inactivity may have independent detrimental effects [5] and few interventions have targeted reductions in screen time in schools, especially in Brazil. A further strength of the program was including strategies that focused on different school contexts and members, an element that is valued in the literature [4, 5, 9, 10]. In particular, PE classes have been a constant (and sometimes the only) focus in interventions and, despite its importance [20], the theoretical aspects [4, 10, 29] of this program warn of a more complex and comprehensive understanding of PA and screen time in young people. Finally, strategies were chosen and structured in order to focus on PA and screen time mediators (see Table 1). This may contribute to the understanding of how the use of different theoretical models of human behavior, including socioecological theory [29], can contribute to active and healthy lifestyle promotion among youth [10, 11].

The *Fortaleça sua Saúde* program was a cluster randomized controlled trial that had a sample size with sufficient statistical power to find the expected moderate effect [11, 16, 35] on the active lifestyle variables, as well as to identify the mediators of these behaviors. The inclusion of students in adolescence was also a strength of the study because similar analyses had only included children [17] and there is a worrying estimate of inactive and unhealthy lifestyles among adolescents [6, 8], including in Brazil [7]. Other strengths of this program were the measurement of potential mediators of PA and screen time [5, 13, 15, 16], and the analysis of whether PA and screen time were predictors of other health factors (e.g., diet and quality of life) and academic performance. A specific evaluation of obese students may indicate the health benefits of this program for a high-risk subgroup of biological, behavioral (objectively-measured), and psychological health problems [4]. Finally, the evaluation of the acceptance, implementation and maintenance of this program may indicate how the *Fortaleça sua Saúde* program strategies may be extended and adapted to the school context [4, 32].

The study’s weaknesses include the relatively short duration of the intervention (one semester, around four months). Although some reviews indicate that short-term interventions can obtain favorable effects on young lifestyles [12], they may not be sufficient to influence all variables. The full-time model is not a comprehensive system in Brazilian schools and this makes it difficult to extrapolate the findings to several schools at this time. Some teachers were aware of which condition in the program the school had been assigned to as a result of the constant communication between the Municipal Education Department and schools. Some staff involved in data collection was also aware of the school’s condition in the program, and the training was focused on minimizing this measurement bias. The lack of blinding can affect the outcomes of the participants in the trial, due to a lack of expectations in a control group or overestimation of results in intervention groups [11]. Finally, despite the use of validated instruments, self-reported measures have measurement bias (e.g., precision, memory, etc.), especially in relation to PA and screen time [6]. The operational and funding conditions of this program made it impracticable to use accelerometers for measuring PA and sedentary time for the whole of the study sample.

We hope the *Fortaleça sua Saúde* program is effective in promoting active and healthy lifestyles and that this represents a program that is well received, implemented, and maintained in the school context. If this occurs, the program’s results could serve as an important basis for public action planning aimed at promoting PA and health among young people, especially from low-and middle-income countries. The results on PA and screen time mediators (from intrapersonal to environmental aspects) may have an additional contribution, guiding the specific and principal focuses on active and healthy lifestyle public policies for young people.

## Additional file

Additional file 1:
**CONSORT 2010 checklist of information to include when reporting a randomised trial*.** (DOC 217 kb)

## Abbreviations

HDI: Human development index
PA: Physical activity
PE: Physical education
PSE: *Programa Saúde na Escola*
WHO: World Health Organization
